# *Aurka* deficiency in the intestinal epithelium promotes age-induced obesity via propionate-mediated AKT activation

**DOI:** 10.7150/ijbs.56477

**Published:** 2021-03-25

**Authors:** Na Sun, Fandong Meng, Jie Zhao, Xueqin Li, Rongqing Li, Jing Han, Xin Chen, Wanpeng Cheng, Xiaoying Yang, Yanbo Kou, Kuiyang Zheng, Jing Yang, Takayuki Ikezoe

**Affiliations:** 1 Jiangsu Province Key Laboratory of Immunity and Metabolism, Affiliated Hospital of Xuzhou Medical University.; 2 Department of Pathogenic Biology and Immunology, Affiliated Hospital of Xuzhou Medical University.; 3 Department of Endocrinology, Affiliated Hospital of Xuzhou Medical University.; 4 National Experimental Demonstration Center for Basic Medicine Education, Xuzhou Medical University, Xuzhou, Jiangsu, China.; 5 The Department of Hematology, Fukushima Medical University, Fukushima, Japan.

**Keywords:** Aurora-A, AKT, obesity, IL-6

## Abstract

Aurora-A kinase, a serine/threonine mitotic kinase involved in mitosis, is overexpressed in several human cancers. A recent study showed that Aurora-A mediates glucose metabolism via SOX8/FOXK1 in ovarian cancer. However, the roles of Aurora-A in metabolic diseases remain unclear. This study found that *Aurka* loss in the intestinal epithelium promoted age-induced obesity and enlargement of lipid droplets in parallel with an increase in infiltrated macrophages in the white adipocyte tissue (WAT) of male mice. Moreover, loss of *Aurka* induced the expression of lipid metabolism regulatory genes, including acetyl-coenzyme A carboxylase 1 (*Acc1*), in association with an increase in the levels of p-AKT in the intestinal epithelium as well as WAT. Blockade of AKT activation reduced the expression of lipid metabolism regulatory genes. In subsequent experiments, we found that the Firmicutes abundance and the levels of short-chain fatty acids (SCFAs) in the gut were dramatically increased in *Aurka^f/+^;Villin^Cre/+^* mice compared with *Aurka^f/+^* mice. Additionally, propionate increased the phosphorylation of AKT* in vitro*. These observations indicated that *Aurka* loss in the intestinal epithelium contributed to gut microbiota dysbiosis and higher levels of SCFAs, especially propionate, leading to AKT activation and lipid metabolism regulatory gene expression, which in turn promoted age-induced obesity.

## Introduction

Obesity has gained widespread recognition as a global pandemic caused by overnutrition and sedentary lifestyles 1. From a public health perspective, obesity is a major risk factor for cancers and metabolic disorders, including nonalcoholic fatty liver disease, diabetes, and cardiovascular disease 2-5. A growing number of studies have indicated that inflammation could play a major role in obesity 6, 7. Additionally, high nutrient content and nutrient absorption appear to be associated with obesity by altering the morphology and function of epithelial cells 8. The mechanisms involved in obesity remain to be fully elucidated, although the incidence of obesity is often attributed to an unhealthy lifestyle.

The mitotic kinase Aurora kinase A (Aurora-A) belongs to the serine/threonine kinase family and controls multiple steps of mitotic progression, including microtubule stability during the G1 phase of the cell cycle, chromosome alignment and segregation, and cytokinesis 9. Multiple studies have suggested that Aurora-A is aberrantly expressed in various types of cancers, including hematopoietic malignancies and colorectal cancer 9. In addition to playing a role in mitosis, Aurora-A is active during G1 and regulates microtubule stability in cooperation with TPX2 and CEP192 10. During neurite extension, Aurora A is also involved in microtubule organization regulation 11. Furthermore, it has been shown that Aurora-A translocates into the nucleus and contributes to distinct oncogenic properties in breast cancer cells by enhancing the breast cancer stem cell (BCSC) phenotype, which is independent of its kinase activity 12. A recent study also showed that Aurora-A translocates into mitochondria and directly interacts with proteins that regulate mitochondrial dynamics, leading to increased mitochondrial fusion 13. Additionally, overexpression of Aurora-A contributes to elevated ATP production in parallel with an increase in the abundance of mitochondrial complex IV 13. Interestingly, the expression of Aurora-A is elevated in human diabetic skin tissue, although the role of elevated Aurora-A in diabetic skin tissues remains unclear 14. These observations imply that Aurora-A's functions beyond its classical mitosis and kinase activity are still largely unexplored.

To further investigate the metabolic function of Aurora-A, we generated Aurora-A conditional knockout (CKO) mice by crossing *Aurka^flox/flox^* (*Aurka^f/f^*) mice with *Villin^Cre/+^* mice, in which Cre-dependent recombination is induced in the intestinal epithelium by embryonic day (E) 12.5 15. We unexpectedly discovered that *Aurka^f/+^;Villin^Cre/+^* mice, though developmentally normal, displayed age-induced obesity and maintained insulin sensitivity well into late adulthood. Further studies revealed that the levels of genes involved in nutrient absorption, including the solute carrier 2A4 (*Slc2a4*) gene, which encodes glucose transporter protein GLUT4, were increased in association with increased expression of p-AKT in *Aurka^f/+^;Villin^Cre/+^* mice.

## Material and Methods

### Generation of conditional *Aurka* knockout mice

Conditional *Aurka* knockout mice were generated as previously described 16. *VillinCre* mice [B6. SJL-Tg (Vil-cre)997Gum/J] were intercrossed with mice carrying loxP-flanked *Aurka* alleles (*Aurka^f/f^*). PCR was utilized to confirm conditional *Aurka* knockout using primers as previously described 16. The mice were bred and maintained strictly according to protocols approved by the Institutional Animal Care and Use Committee at Xuzhou Medical University.

### Metabolic analyses

Body weights were recorded weekly. Metabolic studies were performed as previously described 1. The mice were placed individually in the metabolic chambers of an Oxymax system (Columbus Instruments) and were allowed to acclimate for a 24-hour period. The respiratory exchange ratio (RER) was calculated as the volume of CO_2_ versus the oxygen volume (VCO_2_/VO_2_). To assess energy intake, mice were housed individually, and food consumption was measured. Food consumption data were normalized to body weight measurements. Energy intake in calories was calculated by multiplying the number of grams of normal chow diet consumed daily by 3.93 kcal/gram as described previously 1.

For the glucose tolerance test, 16-hour-fasted mice were injected intraperitoneally with D-glucose (2.5 g/kg body weight). Blood glucose concentrations were examined in tail vein blood samples collected at the designated time points by an Accu-Chek Aviva glucometer (Roche, Basel, Switzerland). Serum triglyceride (TG) and total cholesterol (TC) levels were measured using colorimetric kits (Nanjing Jiancheng Bioengineering Institute, China) based on the protocol recommended by the manufacturer.

### Real-time reverse transcription-polymerase chain reaction (RT-PCR)

RNA was extracted and subjected to real-time RT-PCR as previously described 1. We measured the expression of actin for normalization on a LightCycler 480 (Roche). The primer sets for PCR are shown in Table 1.

### Short hairpin (sh) RNA lentivirus and infection

The shRNA lentivirus used to target Aurora-A was obtained from GeneChem (Shanghai, China). FHC cells purchased from Qingqi (Shanghai, China) were infected with either shcontrol or shAurora-A lentivirus according to the manufacturer's instructions. After 24 hours, LY294002 purchased from Selleckchem (Houston, TX, USA) was added to the indicated wells for 6 hours, and the cells were subjected to immunoblotting.

### Histologic analysis and Oil red O staining

Tissues were fixed in 4% formaldehyde and embedded in paraffin. Sections with a thickness of 4 µm were stained with hematoxylin-eosin (Beyotime Biotechnology, Nantong, Jiangsu, China).

Mouse liver tissues were fixed in 10% neutral formalin for 24 hours and then submerged in 20% sucrose for two days. The liver pieces were frozen in optimal cutting temperature (OCT) solution, and 5-µm-thick tissue sections were cut as previously described 1. Oil red O staining was conducted according to the manufacturer's protocol.

### Multiplexed immunofluorescence staining

Multiplexed staining and multispectral imaging were performed to identify the cell subsets expressing CD11b in WAT or CD3 in intestinal tissues using a PANO 4-Plex IHC Kit (cat 0004100020, Panovue, Beijing, China). Different primary antibodies were sequentially applied, and then horseradish peroxidase-conjugated secondary antibody incubation and tyramide signal amplification were performed. The slides were heated in a microwave after each TSA reaction. The nuclei were stained with 4'-6'-diamidino-2-phenylindole (DAPI, Sigma) after all the antigens had been labeled.

### Immunoblotting

Immunoblotting was performed as previously described 16. Anti-Aurora-A (D3E4Qz, #14475), anti-Acc 1 (#4190), anti-AKT (C67E7, #4691), anti-p-AKT (Ser473, #9271), and anti-PPARγ (C26H12, #2435) antibodies were purchased from Cell Signaling Technology (MA, USA). An anti-SLC7A5/LAT1 (#DF8065) antibody was purchased from Affinity. Anti-β-actin (66009-1-Ig), anti-GLUT4 (66846-1-lg), and anti-GAPDH (60004-1-lg) antibodies were purchased from Proteintech.

### 16S rRNA gene sequence analysis

Genomic DNA amplification and sequencing were performed as described in a previous study 17. Briefly, total DNA was extracted from feces (*n* = 3-5/group) using an E.Z.N.A. Stool DNA Kit (Omega Biotek, Norcross, GA, U.S.) according to manufacturer's protocols.

PCR and custom primers were utilized to amplify the V4-V5 region of the 16S rRNA gene, and sequencing was performed on the Illumina MiSeq platform. 16S rRNA data were analyzed as previously described 17.

### Measurement of fecal short-chain fatty acids (SCFAs)

GC-MS analysis of SCFAs in the feces was performed as previously described to assess SCFA composition 18.

### Enzyme-linked immunosorbent assay (ELISA)

The concentration of IL-1β in the serum was measured using ELISA kits (R&D) according to the manufacturer's instructions. The concentration of IL-6 in the serum was analyzed using an ELISA kit (Jianglai, Shanghai, China) according to the manufacturer's instructions.

### Statistical analysis

Differences were statistically analyzed using Student's t-test or one-way ANOVA followed by Bonferroni post-tests by PRISM statistical analysis software (GraphPad Software, San Diego, CA). The data are presented as the mean ± SEM. Significance is indicated as follows: *P < 0.05; n.s. for not significant.

## Results

### *Aurka* loss in the intestinal epithelium promoted obesity and enlargement of lipid droplets in male mice

**Figure 1:**
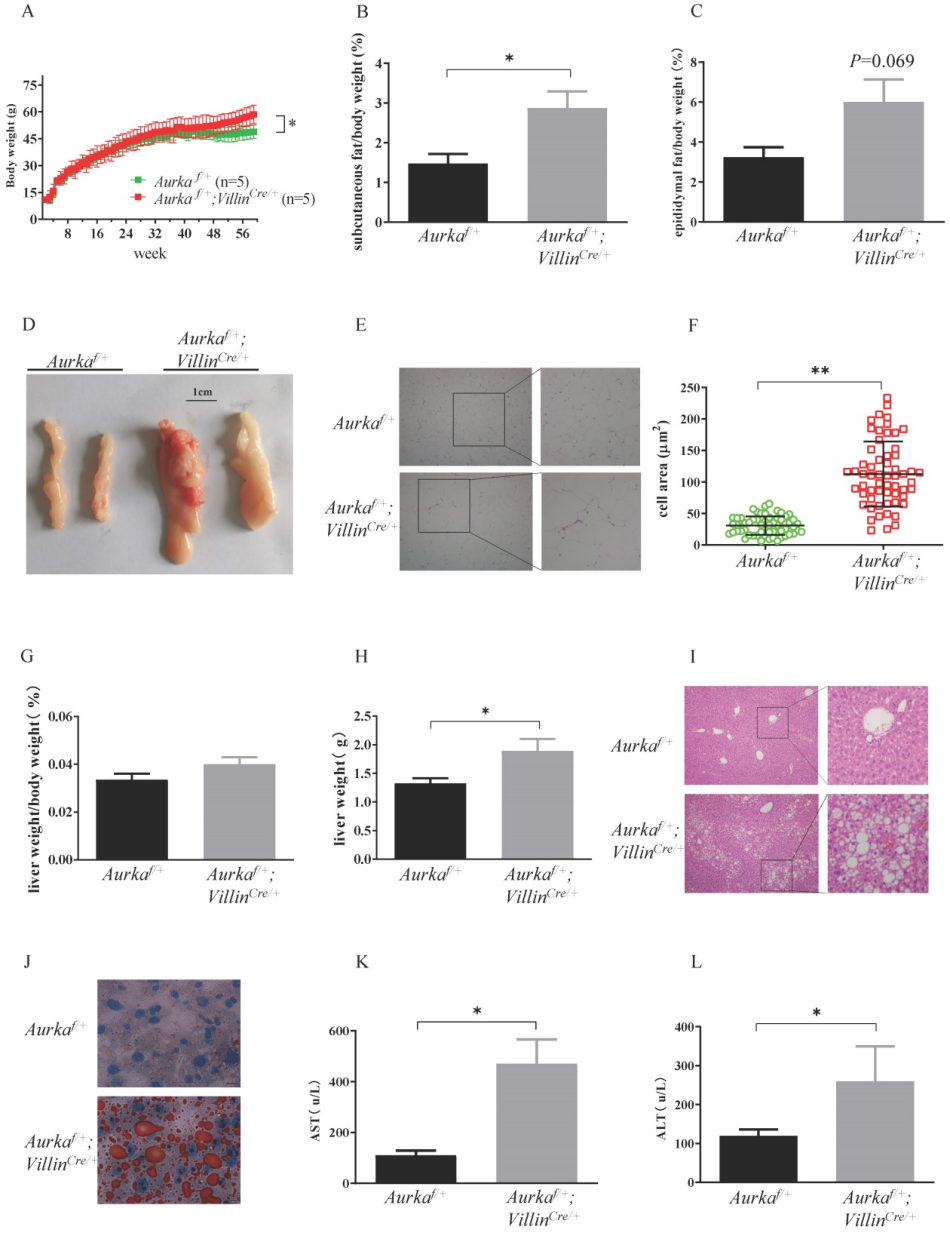
***Aurka* loss in the intestine promoted obesity. (A)** The body weights of *Aurka^f/+^* and *Aurka^f/+^;Villin^Cre/+^* mice were monitored weekly from 2 weeks to 59 weeks (n=5). *, *P* < 0.05.** (B)** The percentage of subcutaneous fat and **(C)** epididymal fat pads compared to body weight. **(D)** Representative images of epididymal fat from *Aurka^f/+^* and *Aurka^f/+^;Villin^Cre/+^* mice.** (E)** Representative photomicrographs of H&E-stained sections of WAT from *Aurka^f/+^* and *Aurka^f/+^;Villin^Cre/+^* mice. **(F)** The cell area was measured with ImageJ (n = 4 mice per group). The graphs show the mean ± SEM of one of two independent experiments. **(G)** At the end of long-term weight monitoring, the livers of the mice were removed and weighed. The data are representative of experiments that included n = 5 livers per group. The results represent the mean ± SEM of liver weight. *, *P* < 0.05. **(H)** Liver weight relative to body weight. **(I, J)** Sections of the livers of *Aurka^f/+^* and *Aurka^f/+^;Villin^Cre/+^* mice were stained by H&E and oil red O of (n = 4 mice per group). The pictures show one of three independent experiments. **(K, L)** Plasma AST and ALT levels were measured. The bar graphs show the mean ± SEM of one of two independent experiments (n = 4 mice per group). *, *P* < 0.05.

*Aurka*-deficient mice exhibited a lethal phenotype. To investigate metabolic function, we generated mice with intestinal epithelium-specific *Aurka* deficiency by crossbreeding *Aurka^f/f^* and *Villin^Cre/+^* mice to obtain *Aurka^f/+^;Villin^Cre/+^* mice. Unexpectedly, when mice were maintained on a normal chow diet, *Aurka^f/+^;Villin^Cre/+^* mice showed significantly greater body weight gain than *Aurka^f/+^* littermates starting at approximately 42 weeks of age (Figure 1A). The weight of subcutaneous fat pads relative to body weight was significantly increased in *Aurka^f/+^;Villin^Cre/+^* mice (Figure 1B). Similarly, the percentage of epididymal fat pads was also increased in *Aurka^f/+^;Villin^Cre/+^* mice compared to that in *Aurka^f/+^* littermates (Figures 1C and 1D). Additionally, as shown in Figures 1E and 1F, the area of white adipocytes in sections of the subcutaneous fat pad was larger in *Aurka^f/+^;Villin^Cre/+^* mice than in *Aurka^f/+^* littermates.

To further clarify the differences in adiposity, we examined liver weight and fat accumulation in the mouse liver, an organ involved in lipid metabolism 1. Liver weight was increased in *Aurka^f/+^;Villin^Cre/+^* mice compared with *Aurka^f/+^* littermates, although the relative size of the livers of *Aurka^f/+^;Villin^Cre/+^* mice were not different from that of the livers of *Aurka^f/+^* mice after normalization to body weight (Figures 1G and 1H). Of note, the livers of 59-week-old *Aurka^f/+^;Villin^Cre/+^* mice exhibited markedly higher lipid droplet accumulation compared than those of *Aurka^f/+^*mice (Figures 1I and 1J). Additionally, the levels of aspartate aminotransferase (AST) and alanine aminotransferase (ALT) were both markedly higher in the serum of *Aurka^f/+^;Villin^Cre/+^* mice than in that of *Aurka^f/+^*mice (Figures 1K and 1L). These observations indicated that loss of *Aurka* in the intestinal epithelium promoted weight gain and enhanced lipid accumulation with age.

### *Aurka* deficiency in the intestinal epithelium affected metabolism

**Figure 2:**
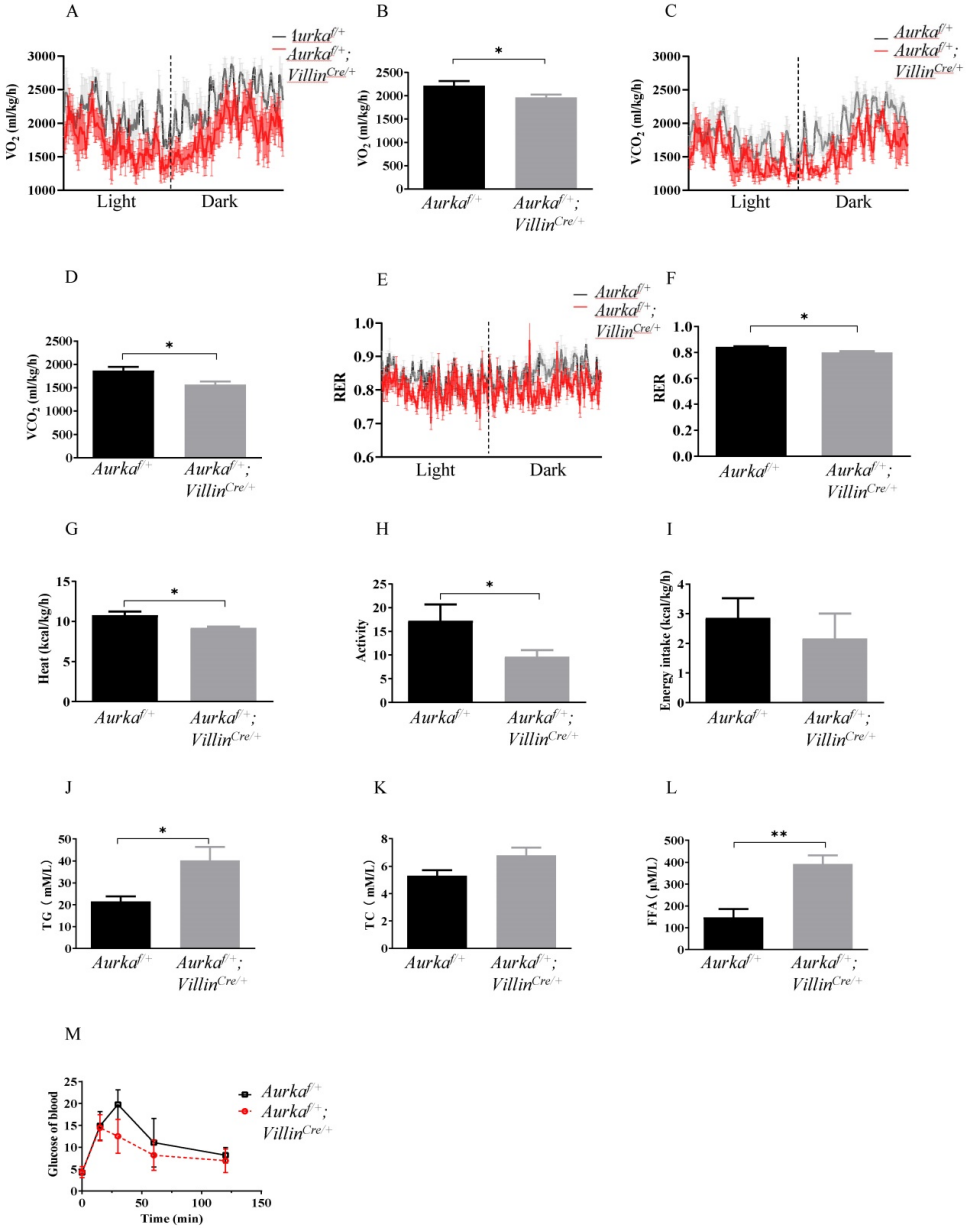
** Metabolism changes were observed in *Aurka^f/+^;Villin^Cre/+^* mice. (A, B, C, D)** O_2_ consumption and CO_2_ production rates at 24 hours were measured via indirect calorimetry using CLAMS under normal chow feeding conditions. The average O_2_ consumption and CO_2_ production rates are shown on the right (n = 4 mice per group). *, *P* < 0.05.** (E)** The respiratory exchange ratio at 24 hours in *Aurka^f/+^* and *Aurka^f/+^;Villin^Cre/+^* mice (n = 4 mice per group). *, *P* < 0.05. **(F, G, H, I)** Histogram showing the average RER, heat, activity, and food intake. The results represent the mean ± SEM (n = 4 mice per group). *, *P* < 0.05. **(J, K, L, M)** Plasma TG, TC, FFA, and blood glucose levels in *Aurka^f/+^* and *Aurka^f/+^;Villin^Cre/+^* mice. The graphs show the mean ± SEM of one of two independent experiments. *, *P* < 0.05; **, *P* < 0.01.

To investigate the underlying mechanisms by which *Aurka* loss in the intestinal epithelium induced obesity, we analyzed metabolic differences between *Aurka^f/+^;Villin^Cre/+^* and *Aurka^f/+^*mice at 59 weeks of age using metabolic cages. The results showed that the *Aurka^f/+^;Villin^Cre/+^* mice exhibited significant decreases in VO_2_, VCO_2,_ and RER (Figures 2A-2F). The *Aurka^f/+^;Villin^Cre/+^* mice also demonstrated a reduction in heat production (Figure 2G). Additionally, a decrease in activity was observed in *Aurka^f/+^;Villin^Cre/+^* mice (Figure 2H). However, there was no significant difference in cumulative food intake between *Aurka^f/+^;Villin^Cre/+^* and *Aurka^f/+^* mice (Figure 2I). Because obesity profoundly affects how triglycerides (TGs) are metabolized in the body 1, we compared serum TG levels between *Aurka^f/+^;Villin^Cre/+^* and *Aurka^f/+^* mice. As we expected, there were significant increases in the serum TG levels but not TC levels (Figures 2J and 2K). We also noted an increase in total cholesterol and FFA levels in the sera of *Aurka^f/+^;Villin^Cre/+^* mice (Figure 2L). Unexpectedly, there was a decreasing trend in glucose levels in *Aurka^f/+^;Villin^Cre/+^* mice (Figure 2M), suggesting that *Aurka* loss in the intestinal epithelium could influence metabolism, thus contributing to obesity rather than glucose tolerance.

### Loss of *Aurka* induced lipid metabolism regulatory gene expression and increased the number of macrophages in WAT

**Figure 3:**
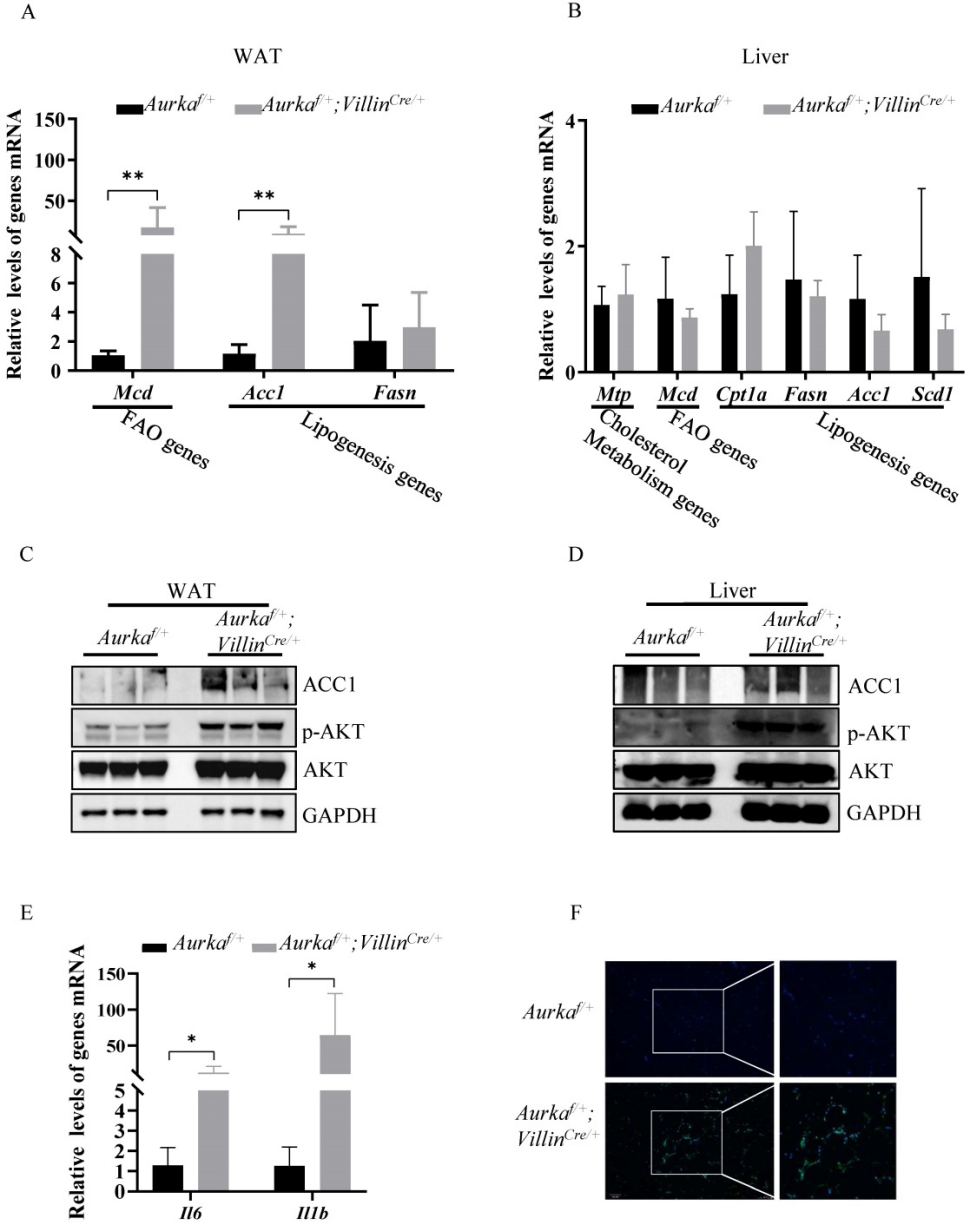
** Lipogenesis, in parallel with increased p-AKT expression, was observed in *Aurka^f/+^;Villin^Cre/+^* mice. (A)** mRNA levels of lipid metabolism-related genes and inflammatory cytokines were measured in WAT from *Aurka^f/+^;Villin^Cre/+^* mice. *, *P* < 0.05. **, *P* <0.01.** (B)** mRNA levels of lipid metabolism-related genes in the liver. **(A, B)** The graphs show the mean ± SEM of one of three independent experiments (n=3 mice per group).** (C, D)** The levels of the indicated proteins were measured in both the WATs and livers of *Aurka^f/+^;Villin^Cre/+^* mice. The pictures are representative one of two independent experiments (n=3 mice per group).** (E)** mRNA levels of inflammatory cytokines in WAT. The histogram shows the mean ± SEM of one of two independent experiments (n=3 mice per group). *, *P* < 0.05. **(F)** CD11b immunostaining of WAT from *Aurka^f/+^ and Aurka^f/+^;Villin^Cre/+^* mice. The pictures show one of three independent experiments.

Since lipogenesis, which occurs predominantly in the liver and adipose tissue, is the primary source of TGs in the liver, we assessed the expression of acetyl-coenzyme A carboxylase 1 (Acc1), one of the critical enzymes involved in promoting de novo lipogenesis 19. We found higher *Acc1* mRNA and protein expression in WAT, but not liver tissues, in *Aurka^f/+^;Villin^Cre/+^* mice than in *Aurka^f/+^* mice (Figures 3A-3D). Moreover, the mRNA levels of *Mcd*, one of the genes involved in FAO 20, in WAT, but not liver tissues, were markedly higher in *Aurka^f/+^;Villin^Cre/+^* mice than in *Aurka^f/+^* mice (Figures 3A and 3B).

Previous studies have indicated that AKT activation plays a significant role in mediating lipid accumulation by indirectly regulating Acc1 expression 21, 22. Therefore, we measured the levels of phosphorylated forms of AKT and found that the levels of p-AKT in WAT and liver tissues were markedly higher in *Aurka^f/+^;Villin^Cre/+^* mice than in *Aurka^f/+^* mice but that the total amount of AKT protein was not different (Figures 3C and 3D).

A growing number of studies found that obesity is characterized by chronic and low-grade inflammation accompanied by an increase in the number of adipose tissue macrophages (ATMs), which play crucial roles in the altered production of several proinflammatory cytokines, such as IL-6 and IL-1β, in the adipose tissues of obese individuals 23-26. The proinflammatory cytokines secreted from ATMs not only repress insulin action 24 but also play an important role in mediating Acc1 expression. Knockout of IL-6 results in decreased expression of Acc1 in mice fed normal chow 27. In addition, IL-6 contributes to AKT activation 28, 29, which also promotes Acc1 expression 21, 22. Therefore, we assessed the mRNA levels of proinflammatory cytokines as well as the percentage of ATMs in WAT. As expected, the mRNA levels of *Il6* and *Il1b* were markedly elevated in WAT from *Aurka^f/+^;Villin^Cre/+^* mice (Figure 3E). Additionally, infiltrated ATMs was dramatically higher in WAT from *Aurka^f/+^;Villin^Cre/+^* mice than in that from *Aurka^f/+^* mice (Figure 3F). Together, these results indicated that *Aurka* loss could contribute to lipid accumulation in parallel with inflammation.

### *Aurka* deficiency promoted nutrient absorption gene expression in the intestine

**Figure 4:**
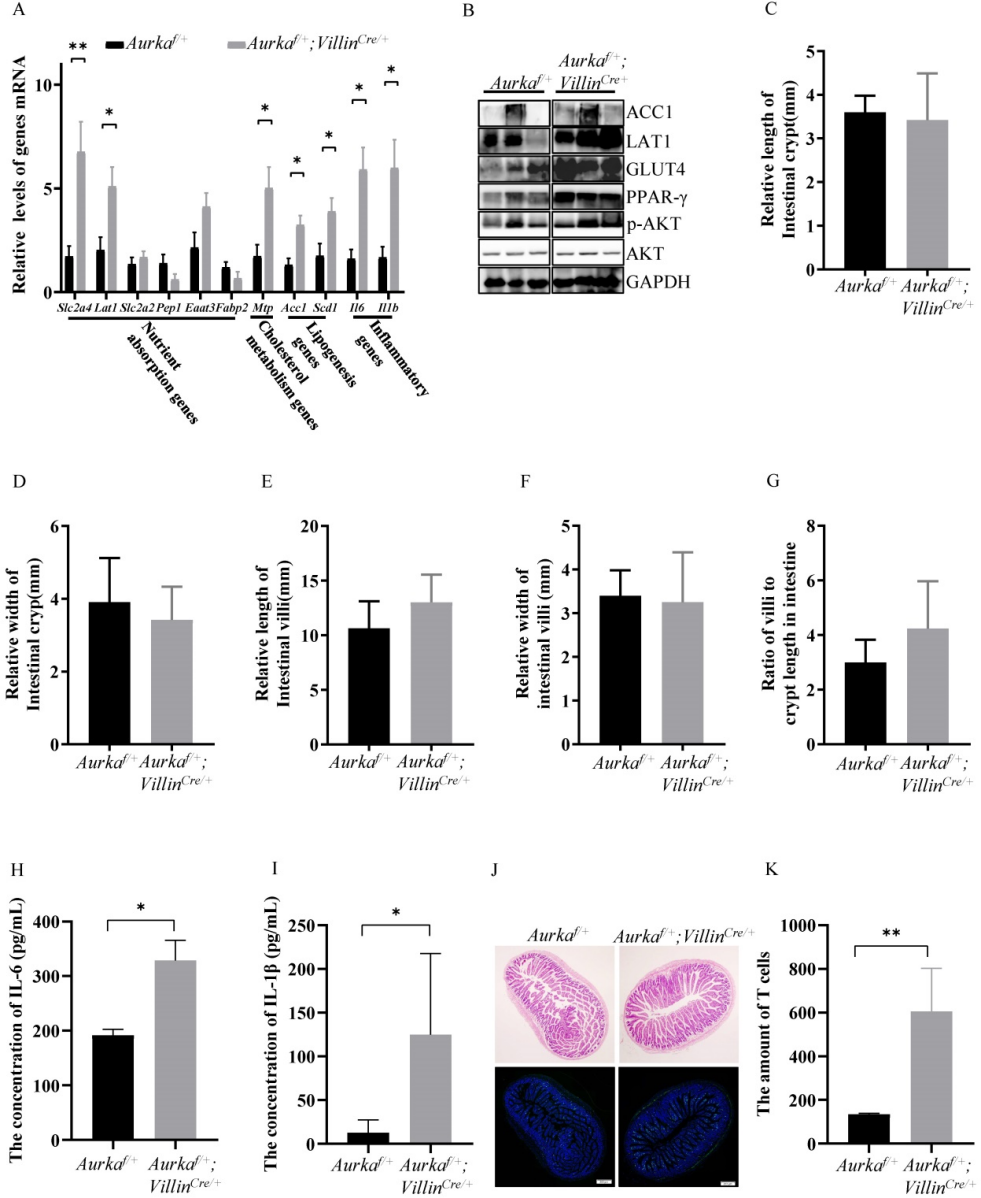
** p-AKT promoted lipid metabolism and nutrient absorption in the intestine of *Aurka^f/+^;Villin^Cre/+^* mice. (A, B)** Lipid metabolism- and nutrient absorption-related mRNA and protein levels of were measured in the intestines of *Aurka^f/+^;Villin^Cre/+^* mice. The graphs show the mean ± SEM of one of three independent experiments. (n=3 mice per group). *, *P* < 0.05. **, *P* <0.01.** (C, D, E, F)** The relative length and width of crypts and villi were measured by ImageJ software. The histogram shows the mean ± SEM. **(G)** The ratio of villi to crypt length in the intestine. **(H, I)** The concentrations of IL-6 and IL-1β in the serum were measured by ELISA. The bar graphs show the mean ± SEM of one of two independent experiments that included five groups of mice. *P <0.05.** (J, K)** H&E staining and CD3 immunostaining of the intestines of *Aurka^f/+^*and *Aurka^f/+^;Villin^Cre/+^* mice. The quantitative data are shown on the right.

Obesity is generally considered the result of an imbalance between food intake, absorption, and energy expenditure, and in this study, no noticeable difference was observed in cumulative food intake between *Aurka^f/+^* mice and obese *Aurka^f/+^;Villin^Cre/+^*mice. We next investigated whether *Aurka* loss could influence nutrient absorption by regulating genes critical for nutrient absorption in *Aurka^f/+^;Villin^Cre/+^*mice. As shown in Figure 4A, the mRNA levels of the* Slc2a4* gene-encoded glucose transporter protein GLUT4, which is critical for mediating glucose uptake and metabolism*,* were significantly increased in intestinal tissues from *Aurka^f/+^;Villin^Cre/+^*mice. Additionally, the levels of solute carrier family 7 member 5 (*SLC7A5*, known as *LAT1*), which belongs to the APC superfamily and forms a heterodimeric amino acid transporter 30, were increased in *Aurka^f/+^;Villin^Cre/+^*mice compared with *Aurka^f/+^*mice. There was a trend toward an increase in the mRNA levels of *Slc2a2* and the excitatory amino acid transporter 3 (*Eaat3*) gene 31, which mediates the uptake of L-glutamate, L-aspartate, and D-aspartate in *Aurka^f/+^;Villin^Cre/+^*mice. Moreover, the mRNA levels of *Mtp, Scd1,* and *Acc1* were markedly increased in *Aurka^f/+^;Villin^Cre/+^* mice (Figure 4A).

Consistently, the protein expression of GLUT4 and ACC1 was enhanced, and the level of p-AKT was increased (Figure 4B). The expression of peroxisome proliferator-activated receptor γ (PPARγ), a member of the PPAR family of nuclear receptors that regulates lipid metabolism and glucose homeostasis 32, was induced in intestinal tissues from *Aurka^f/+^;Villin^Cre/+^* mice (Figure 4B). However, the relative length and width of the intestinal crypts, the relative height and width of intestinal villi, and the ratio of villi to crypt length were almost identical in *Aurka^f/+^;Villin^Cre/+^* mice and *Aurka^f/+^* mice (Figures 4C-4G). These observations further demonstrated that obesity in *Aurka^f/+^;Villin^Cre/+^* mice could result from the elevated expression of nutrient absorption-related genes.

Interestingly, *Il6* and *Il1b* mRNA levels were dramatically increased in the intestinal tissues from *Aurka^f/+^;Villin^Cre/+^* mice (Figure 4A). Consistently, the concentrations of IL-6 and IL-1β were obviously higher in the sera of *Aurka^f/+^;Villin^Cre/+^* mice than in the sera of *Aurka^f/+^* mice (Figures 4H and 4I). Additionally, the number of CD3^+^ cells was markedly increased in *Aurka^f/+^;Villin^Cre/+^* mice compared with *Aurka^f/+^* mice (Figures 4J and 4K), implicating that *Aurka* deficiency in the intestinal epithelium could promote inflammation.

### Loss of *Aurka* in the intestinal epithelium induced gut microbiota dysbiosis in parallel with an increase in the levels of SCFAs

**Figure 5:**
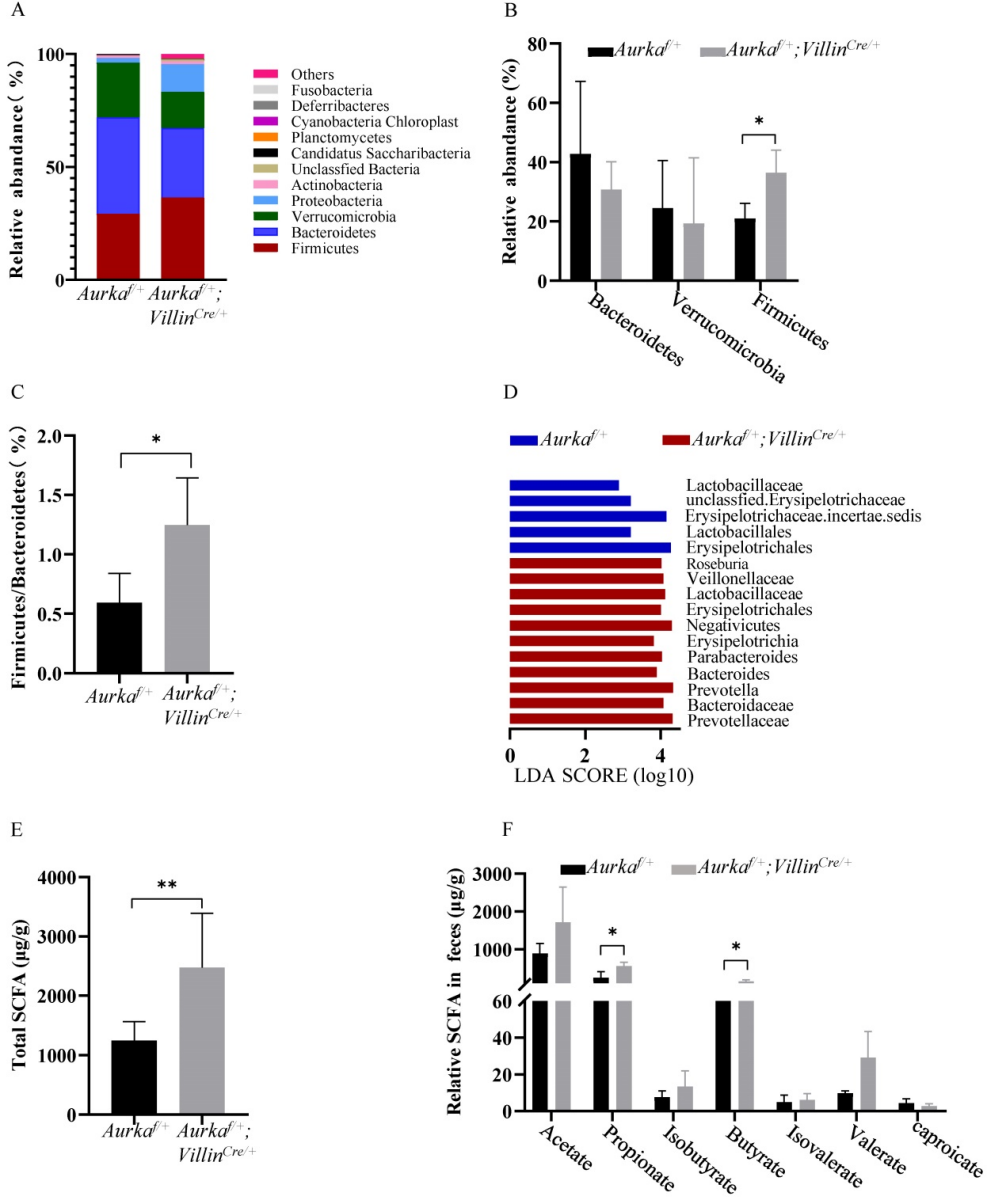
***Aurka* loss resulted in gut microbiota dysbiosis and elevated SCFA levels. (A)** Composition of abundant bacterial phyla. The stacked graph shows the mean ± SEM.** (B)** Comparison of representative taxonomic abundance between *Aurka^f/+^ and Aurka^f/+^;Villin^Cre/+^* mice at the phylum level. The graphs show the mean ± SEM. *, *P* < 0.05.** (C)** The relative taxonomic abundance ratio of Firmicutes to Bacteroidetes (%). *, *P* < 0.05.** (D)** LDA effect size (LEfSe) analysis was utilized to compare the microbiota. the horizontal histogram shows the mean ± SEM. **(E)** Total SCFA production. **, *P* < 0.01.** (F)** Relative levels of the indicated SCFAs. (**E, F**) The results are presented as the mean ± SEM (n=3). *, *P* < 0.05.

Recent studies showed that the gut microbiota improves energy extraction from the diet and modulates plasma levels of lipopolysaccharide (LPS), leading to chronic low-grade inflammation and contributing to obesity 33, 34. We assessed the intestinal microbiota and found that at the phylum level, the abundance of Firmicutes and Proteobacteria was increased in *Aurka^f/+^;Villin^Cre/+^* mice compared to *Aurka^f/+^* mice (Figures 5A and 5B). The relative abundance of Bacteroidetes was decreased in *Aurka^f/+^* mice (Figure 5B). The ratio of Firmicutes/Bacteroidetes was markedly increased (Figure 5C). We next compared the microbiota of *Aurka^f/+^*mice and *Aurka^f/+^;Villin^Cre/+^* mice using linear discriminant analysis (LDA) effect size (LEfSe) analysis. We found that *Aurka* loss in the intestine increased the abundance of Firmicutes at the phylum level and the levels of lower taxa, such as Erysipelotrichia at the class level, Erysipelotrichales at the order level, Erysipelotrichaceae at the family level, and Erysipelotrichaceae incertae sedis at the genus level in the *Aurka^f/+^;Villin^Cre/+^* mice (Figure 5D).

To evaluate whether the levels of SCFAs were increased in *Aurka^f/+^;Villin^Cre/+^* mice compared to *Aurka^f/+^* mice, the fecal levels of SCFAs were measured. Higher levels of total SCFAs, propionate, and isobutyrate were observed in *Aurka^f/+^;Villin^Cre/+^* mice than in *Aurka^f/+^* mice (Figures 5E and 5F). These results collectively suggested that *Aurka* deficiency in the intestine led to gut microbiota dysbiosis and an increase in the levels of SCFAs.

### *Aurka* deficiency-mediated nutrient absorption-related gene transcription depended on SCFA-mediated AKT activation

**Figure 6:**
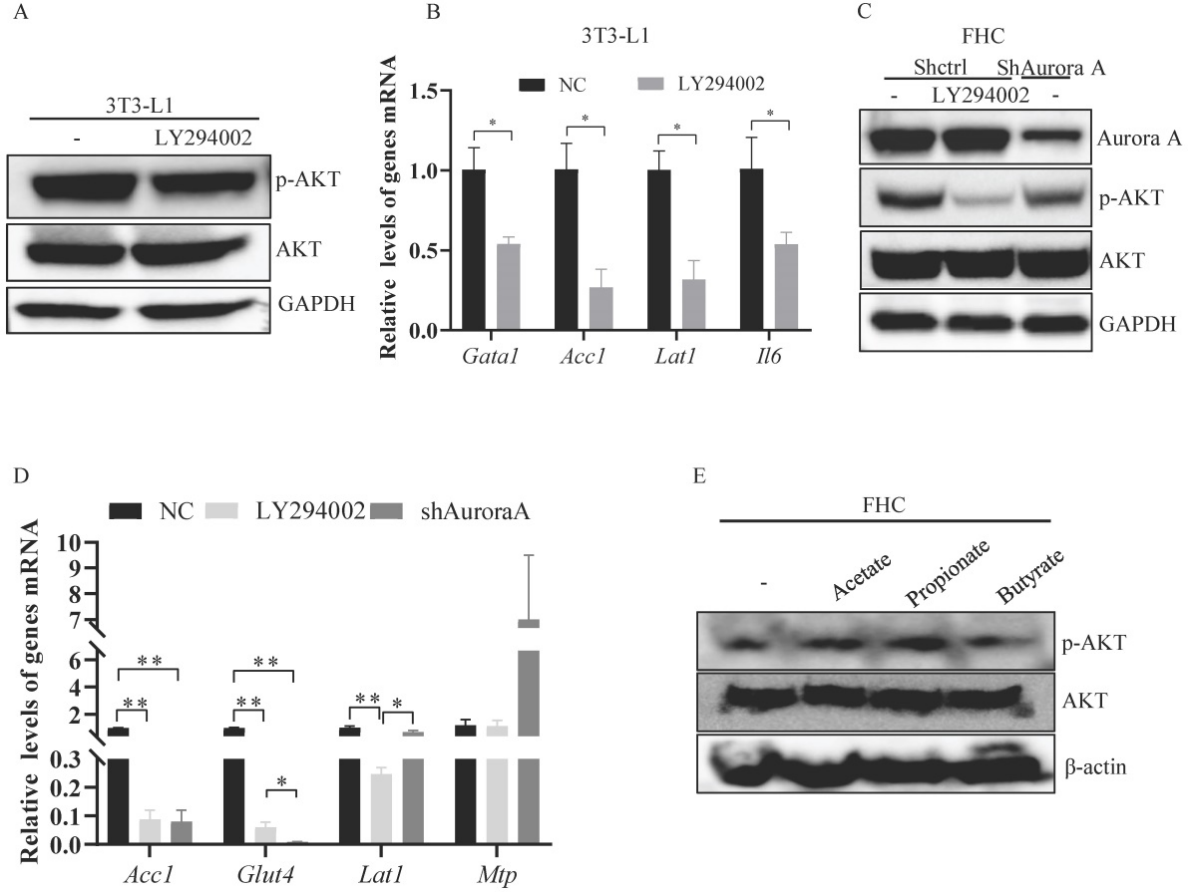
** Propionate-induced activation of AKT. (A)** The expression of the indicated proteins in 3T3-L1 cells was measured by western blot analysis. GAPDH expression is shown as a control. **(B)** Inhibition of the AKT signaling pathway and gene expression were assessed by real-time RT-PCR. The results represent the mean ± SEM of one of three independent experiments. *, *P* < 0.05. **(C)** The expression of the indicated proteins in FHC cells was measured by western blot analysis. GAPDH expression is shown as a control.** (D)** Gene expression was analyzed by real-time RT-PCR. The histogram shows the mean ± SEM of one of three independent experiments in FHC cells. *, *P* < 0.05, **, *P* < 0.01. **(E)** FHC cells were treated with acetate, propionate, or butyrate for 24 hours. The expression of the indicated proteins in these cells was measured by western blot analysis. GAPDH expression is shown as a control.

Previous studies have shown that AKT is critical for the transcription of nutrient absorption-related genes, including *Acc1* 21, 22. Therefore, we treated the murine adipocyte cell line 3T3-L1 and the normal human colon epithelial cell line FHC with LY294002. Upon LY294002 treatment, the level of p-AKT was reduced in 3T3-L1 cells (Figure 6A). *Acc1* and *Lat1* mRNA levels were markedly decreased in 3T3-L1 cells treated with LY294002 (Figure 6B). Similarly, the levels of *Il6* mRNA were also reduced in 3T3-L1 cells treated with LY294002 (Figure 6B). p-AKT expression was dramatically decreased after exposure of FHC cells to LY294002 (Figure 6C). The mRNA levels of *Acc1*, *Glut4,* and *Lat1*, but not *Mtp*, were markedly decreased in FHC cells treated with LY294002 (Figure 6D), suggesting that AKT activation was involved in mediating nutrient absorption-related gene transcription.

To investigate the mechanisms by which AKT was activated in *Aurka^f/+^;Villin^Cre/+^* mice, we knocked down Aurora-A in FHC cells and found that the expression of p-AKT was decreased in FHC cells transfected with shRNA against Aurora-A compared FHC cells transfected with control shRNA (Figure 6C). Additionally, *Acc1*, *Glut4*, and *Lat1* mRNA levels were significantly decreased in FHC cells transfected with shRNA against Aurora-A (Figure 6D). However, *Mtp* mRNA levels were markedly increased in FHC cells transfected with shAurora-A compared with FHC cells transfected with shcontrol (Figure 6D), indicating that AKT could be indirectly activated by *Aurka* loss. SCFAs are involved in mediating the activation of AKT 35. Therefore, we treated FHC cells with the indicated SCFAs to explore whether SCFAs were critical for the activation of AKT in *Aurka^f/+^;Villin^Cre/+^* mice. The levels of p-AKT were elevated after exposure of FHC cells to propionate (Figure 6E). These observations demonstrated that SCFA could mediate activated AKT in the intestine.

## Discussion

**Figure 7:**
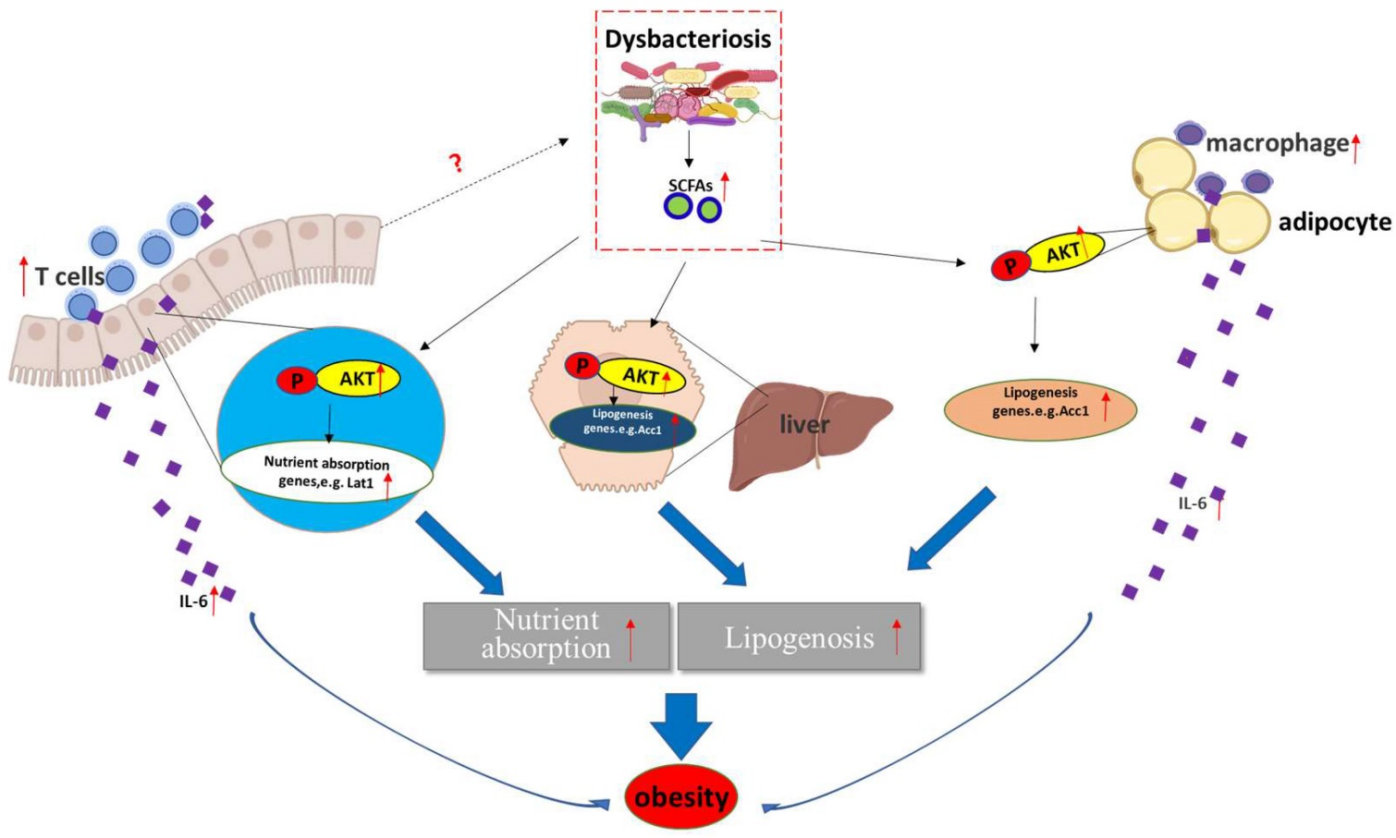
**A hypothetical model by which *Aurka* loss promotes obesity.** *Aurka* loss in the intestinal epithelium leads to gut microbiota dysbiosis and elevated SCFA levels, contributing to the activation of AKT, which in turn promotes nutrient absorption-related gene expression and inflammatory gene transcription, subsequently inducing the development of obesity.

Obese and lean individuals respond differently to nutrients, exhibiting changes in digestion, absorption, and hormone release 8. Additionally, intestinal maintenance and growth are driven by the number of luminal nutrients, with a high nutrient content resulting in increases in cell number, villus length, and crypt depth 8. In this study, we unexpectedly found that deletion of *Aurka* in the intestinal epithelium promoted T cell infiltration without influencing villus length and crypt depth. Notably, *Aurka* loss in the intestinal epithelium increased the abundance of Firmicutes and Proteobacteria as well as the levels of SCFAs (Figure 5), which led to AKT activation, promotion of nutrient absorption and inflammatory gene expression, and subsequent development of obesity (Figure 7).

It has been revealed that the gut microbiota enhances intestinal monosaccharide uptake, contributing to increased de novo lipogenesis and triglyceride accumulation 36, 37. Additionally, by interacting with host epithelial cells, the gut microbiota indirectly regulates energy expenditure and storage 38. A large number of studies suggested that the changes in the relative abundance of Bacteroidetes and Firmicutes are associated with a switch in the metabolic potential of the microbiota to extract energy from food 36, 39, which appears to enable Firmicutes, the “obese microbiota”, to harvest more energy from food. Consistent with a previous mouse study and analysis of the distal gut microbiota in human obesity 40, we found a shift in the gut microbiota shift (a decreased proportion of Bacteroidetes and an increased proportion of Firmicutes) in *Aurka^f/+^;Villin^Cre/+^* mice in the present study (Figures 5A-5D). Unfortunately, how *Aurka* loss in the intestinal epithelium results in a shift in the gut microbiota remains unknown.

In addition to directly interacting with epithelial cells, the gut microbiota indirectly participates in cross-talk with distant organs such as liver and adipose tissue through metabolic products and SCFAs, and mediates obesity 41. It has been shown that SCFAs bind with G-protein-coupled receptors expressed on adipocytes, leading to adipocyte formation 42. Additionally, SCFAs also suppress the synthesis of the hunger-suppressing hormones leptin, peptide YY and glucagon-like peptide 1 43. Consistent with a recent study 35, propionate-induced activation of AKT was shown to be involved in regulating lipid accumulation by indirectly regulating Acc1 expression 17, 18 and nutrient absorption-related gene transcription in a human FHC cell line (Figure 6E). Consistently, the levels of Acc1 were increased in the WAT, liver and intestine (Figures 3C, 3D and 4B). Furthermore, the mRNA levels of nutrient absorption-related genes, including *Slc2a4*, *Lat1*, and* Eaat3,* were increased in* Aurka^f/+^;Villin^Cre/+^* mice compared with *Aurka^f/+^*mice (Figure 4A). However, inhibition of AKT by LY294002 dramatically decreased the mRNA levels of these genes (Figure 6D). These observations suggested that loss of *Aurka* in the intestinal epithelium could promote obesity at least partially via SCFA-mediated AKT activation. In addition, gut microbiota dysbiosis resulted in inflammation, as indicated by the presence of ATMs and infiltrated CD3^+^ T cells in the WAT and intestine, respectively (Figures 3F and 4J), which led to increases in the mRNA levels and concentrations of proinflammatory cytokines such as IL-6 and IL-1β in the sera of *Aurka^f/+^;Villin^Cre/+^* mice (Figures 3E, 4A, 4H and 4I). Previous studies have demonstrated that IL-6 also induces AKT activation 44. This implies that activated AKT could also be mediated by upregulation of IL-6 expression in *Aurka^f/+^;Villin^Cre/+^*mice.

Taken together, these data suggest that *Aurka* deficiency in the intestinal epithelium promoted age-induced obesity, resulting in gut microbiota dysbiosis and elevated SCFA levels and leading to AKT activation, which subsequently contributed to the upregulation of nutrient absorption-related gene expression.

## Figures and Tables

**Table 1:** Real-time RT-PCR primers

Gene	Direction	Primer
*Pept1* (mouse)	Forward	5'- AACTGTGGCGGTCGGCAATAT -3'
	Reverse	5'-CTGTGCTTCAATCTCTGCTGGGT-3'
*Il6* (mouse)	Forward	5'- CCGGAGAGGAGACTTCACAG -3'
	Reverse	5'- CCACGATTTCCCAGAGAAC -3'
*Lat1* (mouse)	Forward	5'-GCACCACCATCTCCAAGTCAGG-3'
	Reverse	5'-GAATGATGAGCAGCTCGATCCA-3'
*Eaat3* (mouse)	Forward	5'-ATTTTCCATTTTGACCTCATCTCCA -3'
	Reverse	5'-TTCCCCTAAACCCCACAACTATCTT-3'
*Il1b* (mouse)	Forward	5'-CAGGCAGGCAGTATCACTCA -3'
	Reverse	5'-TGTCCTCATCCTGGAAGGTC -3'
*Mtp* (mouse)	Forward	5'-CTTCCGCACTTTCCGAGATG-3'
	Reverse	5'-TCAAAGCCCAGGACTGTCAT-3'
*Fabp2* (mouse)	Forward	5'-TGTTGTGTTTGAGCTCGGTG-3'
	Reverse	5'-TTTCCCTCAATGGTCCAGGC-3'
*Slc2a2* (mouse)	Forward	5'- CTGCACCATCTTCATGTCGG-3'
	Reverse	5'- ACCTGGCCCAATCTCAAAGA -3'
*Glut4* (mouse)	Forward	5'- CCGAAAGAGTCTAAAGCGCC-3'
	Reverse	5'- GCTCTCTCTCCAACTTCCGT -3'
*Scd1* (mouse)	Forward	5'-CTTCCTCCTGAATACATCCCTCC-3'
	Reverse	5'- CTCCATCCCATCTAGCACAACCT -3'
*Acc1* (mouse)	Forward	5'- TGCTGGATTATCTTGGCTTCA-3'
	Reverse	5'- CCCGTGGGAGTAGTTGCTGTA -3'
*Il6* (human)	Forward	5'-TACATCCTCGACGGCATCTC -3'
	Reverse	5'-AGTGCCTCTTTGCTGCTTTC -3'
*Mtp* (human)	Forward	5'-GGACGTCAAGAACATCCTGC-3'
	Reverse	5'-CGACGGACAATTTTGCTTGC-3'
*Il1b* (human)	Forward	5'-ACGATGCACCTGTACGATCA-3'
	Reverse	5'-GGAGGTGGAGAGCTTTCAGT-3'
*Pparγ* (human)	Forward	5'-GACCACTCCCACTCCTTTGA-3'
	Reverse	5'-TCTGCAACCACTGGATCTGT-3'
*Acc1* (human)	Forward	5'-ACGGCATCATTAACTGGGGA-3'
	Reverse	5'-TATCCACATCCGGGGCAATT-3'
*Glut4* (human)	Forward	5'-CTGGCCATCATCATCTCCCT-3'
	Reverse	5'-GTGGACAGGGTGGTGAAGTA-3'
*Pparγ* (mouse)	Forward	5'-TCTTAACTGCCGGATCCACA-3'
	Reverse	5'-GCATTGTGAGACATCCCCAC-3'
*Scd1* (mouse)	Forward	5'-CTTCCTCCTGAATACATCCCTCC-3'
	Reverse	5'- CTCCATCCCATCTAGCACAACCT -3'
*18S* (human)	Forward	5'-AAACGGCTACCACATCCAAG -3'
	Reverse	5'- CCTCCAATGGATCCTCGTTA-3'
